# Fatty acids modulate the expression levels of key proteins for cholesterol absorption in Caco-2 monolayer

**DOI:** 10.1186/s12944-018-0675-y

**Published:** 2018-02-20

**Authors:** Fang Yang, Guoxun Chen, Meihu Ma, Ning Qiu, Lingjiao Zhu, Jing Li

**Affiliations:** 1 National Research and Development Center for Egg Processing, College of Food Science and Technology, Huazhong Agricultural University, 430070 Wuhan, Hubei People’s Republic of China; 2 School of Laboratory Medicine, Hubei University of Chinese Medicine, 1 Huangjia Lake West Road, Wuhan, 430065 China; 3 Department of Nutrition, University of Tennessee at Knoxville, Knoxville, TN USA

**Keywords:** Fatty acids, Intestinal cholesterol absorption, Caco-2 monolayer transport, NPC1L1

## Abstract

**Background:**

Fatty acids have been shown to modulate intestinal cholesterol absorption in cells and animals, a process that is mediated by several transporter proteins. Of these proteins, Niemann-Pick C1-Like 1 (NPC1L1) is a major contributor to this process. The current study investigates the unknown mechanism by which fatty acids modulate cholesterol absorption.

**Methods:**

We evaluated the effects of six fatty acids palmitic acid (PAM), oleic acid (OLA), linoleic acid (LNA), arachidonic acid (ARA), eicosapentaenoic acid (EPA) and docosahexaenoic acid (DHA) on cholesterol uptake and transport in human enterocytes Caco-2 cells, and on the mRNA expression levels of NPC1L1, others proteins (ABCG5, ABCG8, ABCA1, ACAT2, MTP, Caveolin 1, Annexin-2) involved in cholesterol absorption, and SREBP-1 and SREBP-2 that are responsible for lipid metabolism.

**Results:**

The polyunsaturated fatty acids (PUFAs), especially for EPA and DHA, dose-dependently inhibited cholesterol uptake and transport in Caco-2 monolayer, while saturated fatty acids (SFAs) and monounsaturated fatty acids (MUFAs) had no inhibitory effects. EPA and DHA inhibited cholesterol absorption in Caco-2 monolayer might be caused by down-regulating NPC1L1 mRNA and protein levels, which were associated with inhibition of SREBP-1/− 2 mRNA expression levels.

**Conclusion:**

Results from this study indicate that functional food containing high PUFAs may have potential therapeutic benefit to reduce cholesterol absorption. Further studies on this topic may provide approaches to control lipid metabolism and to promote health.

**Electronic supplementary material:**

The online version of this article (10.1186/s12944-018-0675-y) contains supplementary material, which is available to authorized users.

## Background

Dietary cholesterol contributes to cholesterol homeostasis. An excessive intake of dietary cholesterol may lead to an increase in plasma cholesterol levels, particularly low-density lipoprotein (LDL) cholesterol level, and atherosclerotic cardiovascular diseases (CVDs) risk [1]. Because the plasma cholesterol level is partly influenced by diets, the types of dietary fat may impact the cholesterol level and heart diseases. Research work for the past 60 years has indicated the impact of fatty acids on cholesterol metabolism. Diets high in saturated fatty acids (SFAs) often lead to increased plasma total cholesterol and low-density lipoprotein (LDL) cholesterol concentrations compared to a diet high in monounsaturated fatty acids (MUFAs) and polyunsaturated fatty acids (PUFAs) [2–7]. It has been shown previously that the intake of MUFAs lowered LDL-cholesterol without affecting high-density lipoprotein (HDL) cholesterol [5, 6, 8]. Some groups even reported increases in the HDL level [3, 4]. In addition, elevation of MUFAs intake rather than replacement of SFAs by MUFAs also led to a decrease in total and LDL cholesterol levels [9]. On the other hand, it has been shown that the dietary intake of n-3 PUFAs increases LDL particle size [10, 11] and exerts lipid-lowering effects; in particular, reductions in plasma cholesterol levels have been described in humans and animals models, although consistent results are lacking [12, 13]. Moreover, in secondary prevention trials, n-3 PUFAs from fish or supplements reduced premature mortality and sudden death in individuals with a history of myocardial infarction [13] and coronary artery disease [14]. The primary prevention studies have shown the benefits of high-dose n-3 PUFAs supplements on metabolic risk factors; for instance, reductions in triacylglycerol levels have been reported [5, 15].

Plasma cholesterol homeostasis in the body is controlled mainly by endogenous synthesis, intestinal absorption, and hepatic excretion. When it focuses on cholesterol intestinal absorption, Chen et al. found out that cholesterol absorption in the thoracic duct lymph of rats with a drainage catheter in the left thoracic lymphatic channel was significantly reduced in the presence of menhaden oil or fish oil concentrate compared to that of corn oil in 1987 [16]. Furthermore, n-3 PUFAs have been shown to decrease cholesterol absorption in animal models when a high amount of cholesterol is present in the diet [17–19]. Dietary cholesterol is absorbed from micelles with fatty acids and phospholipids in the proximal parts of the small intestine, re-esterified into cholesteryl esters for the assembly into lipoproteins, and transported to the lymph and then to the circulations. It has been shown that cholesterol transport-related proteins play important roles in cholesterol metabolism by regulating the cholesterol absorption in enterocytes. Key proteins involved in dietary cholesterol uptake in the enterocytes have been identified during the past few years, i.e., the cholesterol uptake transporter, Niemann-Pick C1-Like 1 (NPC1L1) [20]; the cholesterol efflux transporters, ATP-binding cassette (ABC) proteins ABCG5 and ABCG8 [21], ABCA1; other regulators such as acetyl-Coenzyme A acetyltransferase 2 (ACAT2), microsomal triglyceride transfer protein (MTP), caveolin 1, annexin A2; and cholesterol transcription factors sterol regulatory element-binding proteins (SREBP-1 and SREBP-2) [22].

In the lumen of the small intestine, unesterified free cholesterol from dietary intake and biliary secretion is solubilized into micelles, which containing bile acids, fatty acids, phospholipids, and monoacylglycerols. NPC1L1, which functions as a sterol transporter to mediate intestinal cholesterol absorption crossing intestinal brush border membranes into enterocytes and counterbalances hepatobiliary cholesterol excretion [20]. NPC1L1 is an essential intestinal component of cholesterol absorption and may be transcriptionally regulated by cellular cholesterol content through SREBP-2, a transcription factor that binds to the two sterol regulatory elements in the human NPC1L1 promoter and increases NPC1L1 promoter activity in Caco-2 cells [20, 23]. Recent findings suggest that NPC1L1 deficiency also prevents diet-induced hepatic steatosis and obesity in addition to reducing plasma cholesterol [24, 25]. The majority of absorbed and endogenously synthesized cholesterol is transported to the endoplasmic reticulum, where it is converted to cholesterol ester by ACAT2 and is then assembled into chylomicrons in a MTP-dependent manner for secretion into the circulation via the lymphatic system [22]. Furthermore, ABCG5 and ABCG8 represent apical sterol export pumps that promote active efflux of cholesterol and plant sterols from enterocytes back into the intestinal lumen for excretion [21]. Cholesterol may also be transported into the circulation as a constituent of HDL via localized ABCA1 at the basolateral membrane of enterocytes [22]. In vitro studies demonstrated that oleic acid (OLA) [26] and PUFAs [27, 28] in Caco-2 cells significantly down-regulated the expression of NPC1L1 to decrease cholesterol absorption [28]. Although the mechanism involved in the cholesterol-lowering effect of unsaturated FAs (UFAs) is not fully understood, activation of transcription factor SREBP 1/2 appears to be involved [29]. The absorption efficiency of cholesterol is most likely determined by the net balance between influx and efflux of intraluminal cholesterol molecules across the brush border of the enterocytes. Although limited knowledge support a direct effect of different types of fatty acids on cellular cholesterol transporters in the gut lumen and on intestinal cholesterol absorption, it has been demonstrated that PUFAs in the gut lumen is able to affect cholesterol uptake. However, it is not clear how this biological effect might be mediated at the cellular or molecular level.

The human epithelial Caco-2 cell monolayer model has been used commonly to study the intestinal absorption of drug candidates and mechanisms of drug transport across the epithelial layer [30, 31]. Previous studies have examined cholesterol distribution in apical medium, basolateral medium and cells [32–34]. However, cholesterol transport under the influence of different types of fatty acids across the Caco-2 monolayer has not been done. Both passive and active parallel transport routes can be studied in this advantageous model system. All these can lead to the improvement of screening candidates for regulating cholesterol absorption, which potentially have therapeutic benefits and clinical implications. Therefore, the present study was performed to investigate the effects of six fatty acids, palmitic acid (PAM), oleic acid (OLA), linoleic acid (LNA), arachidonic acid (ARA), eicosapentaenoic acid (EPA) and docosahexaenoic acid (DHA), as shown in Table 1, on cholesterol uptake and transport across the Caco-2 monolayer, and the possible regulatory mechanisms.

**Table 1:** Structure of six fatty acids with a methyl end and a carboxyl end^a^

	Methyl end Carboxyl end	Saturation	△ − characteristics
Palmitic acid (PAM)		Saturate	C16:0
Oleic acid (OLA, ω-9)		Monoene	C18:1 Δ9
Linoleic acid (LNA, ω-6)		Polyene	C18:2 Δ9, 12
Arachidonic acid (ARA, ω-6)		Polyene	C20:4 Δ5, 8, 11, 14
Eicosapentaenoic acid (EPA, ω-3)		Polyene	C20:5 Δ5, 8, 11, 14, 17
Docosahexaenoic acid (DHA, ω-3)		Polyene	C22:6 Δ4, 7, 10, 13, 16, 19

^a^ Palmitic acid is a trivial name for a saturated fatty acid with 16 carbon atoms and no double bonds (C16:0). Oleic acid has 18 carbon atoms and one double bond in the ω-9 position (C18:1 ω-9), whereas Linoleic acid has 18 carbon atoms and two double bonds in the ω-6 position (C18:2 ω-6), *ARA* Arachidonic acid, *EPA* eicosapentaenoic acid, *DHA* docosahexaenoic acid, with multiple double bonds, are represented as C20:4 ω-6, C20:5 ω-3 and C22:6 ω-3. This numerical scheme is the systematic nomenclature most commonly used. It is also possible to describe fatty acids systematically in relation to the acidic end of the fatty acids; symbolized Δ (Greek delta) and numbered 1. All unsaturated fatty acids are shown with *cis* configuration of the double bonds

## Methods

### Materials

PAM, OLA, LNA, ARA, EPA, DHA, L-α-phosphatidylcholine, cholesterol, sodium taurocholate, 1-oleoyl-rac-glycerol (monoolein), 4-(2-hydroxyethyl)-1-piperazineethanesulfonic acid (HEPES) sodium salt 99%, non-essential amino acids, lucifer yellow, dimethyl sulfoxide (DMSO) and all Hank’s Balanced Salt Solution (HBSS) buffer constituents were purchased from Sigma-Aldrich (Bornem, Belgium). Cholestyramine was purchased from Sequoia Research Products Ltd. (Pangbourne, UK). [1, 2-^3^H (N)]-cholesterol (1.85 TBq/mmol) was purchased from Perkin Elmer (NEN, USA). [^14^C]-sodium taurocholate (1.89 Gbq/mmol) was purchased from Amersham International (Buckinghamshire, United Kingdom).

Caco-2 cells were purchased from American Tissue Culture Collection (Rockville, USA). Dulbecco’s modified Eagle medium (DMEM), fetal bovine serum (FBS), 100 × nonessential amino acids, 100 × penicillin and streptomycin, 0.25% trypsin with ethylenediaminetetraacetic acid (EDTA) and BSA (Bovine serum albumin) were purchased from Thermo Scientific HyClone (Logan, USA). Transwell permeable polycarbonate inserts (0.4 μm) and 12-well cell culture plates were obtained from Corning Costar (New York, USA). Primers used in quantification of mRNA by PCR were provided by Sango Biotech (Shanghai, China). Rabbit NPC1L1 monoclonal antibody was purchased from Epitomics (5386-1, California, USA).

### Caco-2 cell culture and experiment preparation

Caco-2 cells were cultured as described with some modifications [35]. Cells (passage numbers 41–51) were grew in 25 cm^2^ plastic flasks at 37 °C in a humidified atmosphere with 5% CO_2_ in high glucose DMEM with 20% (*v*/v) heat-inactivated FBS, 1% L-glutamine, 1% non-essential amino acids, 20 mmol/L HEPES, 100 U/ml penicillin and 100 μg/ml streptomycin (pH 7.4). Once the flasks reached 80% confluency, the cells were split and seeded at a density of 1.25 × 10^4^ cell/cm^2^ onto polycarbonate micropore membranes (0.4 μm pore size, 12 mm diameter) inserted into transwells. Cells were fed every other day and were used 21 days after seeding.

Delipidized FBS was prepared according to the method of Gibson et al. [36]. In essence, 20 g of thixotropic gel powder (Cab-o-sil, Fibre Glast) was added to 1 L FBS and stirred overnight at 4 °C. The mixture was then centrifuged at 12,000×g and 4 °C for 1 h. The supernatant was sequentially filtered through a 0.20 μm filter (25 mm, GD/X, Whatman, Inc., NJ, USA) and kept at 4 °C until being used. The composition and preparation of mixed micellar solutions were performed according to previous studies [6]. Micellar solutions with 0.1% (*v*/v) DMSO were as follows: 2 μCi/mL [1,2-^3^H (N)]-cholesterol, 100 μmol/L cholesterol, 1 mmol/L oleic acid for control or 0.1, 0.5, 1.0 mmol/L fatty acid (palmitic acid (PAM, C16:0) / oleic acid (OLA, C18:1) / linoleic acid (LNA, C18:2) / arachidonic acid (ARA, C20:4) / eicosapentaenoic acid (EPA, C20:5) or docosahexaenoic acid (DHA, C22:6), 0.5 mmol/L monoolein, 6.6 mmol/L sodium taurocholate, and 0.1 mmol/L soy phospatidylcholine (PtdCho). The specific activity of [1,2-^3^H (N)]-cholesterol in this micellar solutions was 4.44 × 10^7^ dpm/μmol. The tracer with the same specific activity was used in the cholesterol uptake and transport experiments. To prepare the micellar solution, the appropriate amounts of above mentioned reagents were dissolved in culture medium (uptake experiment) or HBSS buffer (transport experiment), and the concentrations of fatty acids were indicated in figure legends and table notes. The micellar solutions were subjected to sonication, then passed through a 0.20 μm filter (25 mm, GD/X, Whatman, Inc., NJ, USA) and kept at 37 °C until being used.

In cholesterol transport experiments, Caco-2 monolayer model was established in 12-well transwell inserts, as monitored by morphology, alkaline phosphatase activity and monolayer permeability according to the method of Hubatsch [37]. Morphology of Caco-2 cell monolayer during and after differentiation was monitored by scanning electron microscope and transmission electron microscopy. Cells after the 21-day culture were fixed in 2.5% glutaraldehyde treatment for 2 h, and then post-fixed for 30 min in 1% osmium tetroxide buffer and incubated in freshly made 1% carbohydrazide for 30 min. The fixed cells were rinsed three times with distilled water for over 15 min each time. The bottom of cell culture dishes was cut to fit the critical point dryer. Then, after a series of alcohol dehydration, dried in critical point dryer and sputter coat with 1-2 nm gold-palladium. Then, the cell ultrastructure was observed under scanning electron micrograph (JSM-6390/LV). For transmission electron microscopy, cells were fixed in 2.5% glutaraldehyde solution for 4 h and then post-fixed for 2 h in 1.33% osmium tetroxide buffered with 0.1 mol/L cacodylate. Then after a series of alcohol dehydration, propylene oxide/resin 2:1 infiltration, resin embedding and sectioning, the cell ultrastructure was observed under transmission electron microscopy (Hitachi H-7100). Alkaline phosphatase activity of the Caco-2 monolayer was measured using the Alkaline Phosphatase Activity Assay Kit (CBA-301, Cell Biolabs, Inc., CA, USA). The integrity of each monolayer of differentiated cells was monitored by *P*_app_ value of lucifer yellow and the transepithelial electrical resistance (TEER) with a Millicel ERS-2 voltmeter (Millipore, MA, USA), which was calculated as in Eq. (1).

1 $$ TEER\kern0.5em \left(\Omega \kern0.5em {cm}^2\right)=\left[\mathrm{TEER}\left(\Omega \right)-{\mathrm{TEER}}_{\mathrm{background}}\left(\Omega \right)\right]\times \mathrm{area}\left({\mathrm{cm}}^2\right) $$

In Eq. (1), TEER (Ω) is the electrical resistance across the Caco-2 monolayer directly read from the ERS-2 epithelial voltmeter, the TEER_background_ (Ω) is the electrical resistance across the insert only (without cells) and the area (cm^2^) is the area of the insert, 1.12 cm^2^.

### Fatty acids oxidation and cytotoxicity experiments

Fatty acids oxidation in stock solutions at concentrations of 0.1, 0.5, 1.0 mmol/L was measured with a thiobarbituric acid reagent using malondialdehyde (MDA) as a standard [38]. Oxidized solutions of fatty acid were generated as described previously [39] and were used as positive control. Results are expressed as MDA/FA (μM/μM). The 3-(4, 5-dimethylthiazole-2-yl)-2, 5-diphenyltetrazolium bromide (MTT) was used to determine the cytotoxicity of phospholipids in Caco-2 cells with the methods of Mosmann [40]. The quantity of formazan (presumably directly proportional to the number of viable cells) is measured by recording changes in absorbance at 570 nm using a plate reading spectrophotometer.

### Cholesterol uptake and transport experiments after adding fatty acids

The cholesterol uptake was examined with the methods of Ikeda [33]. Cholesterol micellar solutions (1 mL) with 2 μCi/mL [1,2-^3^H (N)]-cholesterol, 100 μmol/L cholesterol, 1 mmol/L OLA for Control or 0.5 mmol/L fatty acid (PAM / OLA / LNA / ARA / EPA / DHA), 0.5 mmol/L monoolein, 6.6 mmol/L sodium taurocholate, and 0.1 mmol/L soy PtdCho in cultural media containing the delipidized FBS were added to 12-well plates where Caco-2 cells had grown for 21 days. The cells were then incubated in the micellar medium for 60 min and 120 min, respectively. At the end of the incubation, the medium was removed, and the cells were detached from the culture dishes with 0.25% trypsin treatment, centrifuged at 1500×*g* and 4 °C for 5 min to obtain the cell pellets. The cell pellets were dissolved in 0.5 mL of 0.1 mol/L NaOH, and an aliquot of 0.1 mL of the lysate was used for analysis of radioactivity. As Caco-2 cells synthesize cholesterol [22], radioactive cholesterol was commonly introduced to investigate cellular cholesterol uptake and transport as shown by others [33, 34, 41]. Therefore, we used radiolabeled cholesterol as a tracer to differentiate the endogenous and exogenous cholesterol in the absorption and transport experiment. Since a small portion of the radioactive cholesterol was absorbed and then secreted back to the cultural medium [22], the radioactivity in the medium was also measured as part of the total radioactivity. The samples were mixed with an equal volume (100 μL/100 μL) of scintillation liquid mix (5 g PPO and 0.5 g POPOP dissolved in 100 mL xylene), and the counts per minute (cpm) were detected by using the 1450 MicroBeta TriLux liquid scintillation counter (Perkin Elmer, Waltham, Massachusetts, United States). The total protein of Caco-2 cell lysates was quantified by BCA Protein Assay Kit (Beyotime, Shanghai, China). The machine was calibrated by using a series of standards containing different concentrations of ^3^H-cholesterol in HBSS solutions, which had the known radioactive decay per minute (dpm) values. The detection efficiency of liquid scintillation counter was calculated from the known standard ^3^H-cholesterol activity (dpm), which is (cpm_standard_-cpm_bcakground_)/dpm × 100%. In this paper, the detection efficiency of liquid scintillation counter ranged from 0.57 to 0.62. The radioactive cpm values of the samples were converted into dpm according to the detection efficiency of liquid scintillation counter and presented as dpm/mg total protein. Since the uptake rates of 2 μCi/mL [1,2-^3^H (N)]-cholesterol tracer and 100 μmol/L cholesterol in Caco-2 cells were the same, so the rate of uptake and transport of 100 μmol/L cholesterol from micelles could be calculated by the rate of radio labeled cholesterol tracer. In order to obtain the exact cholesterol uptake amount, the results were eventually expressed as pmol cholesterol/mg protein based on the radioactive cholesterol in cells and medium derived from total added cholesterol (100 μmol/L).

Transport procedures for the determination of the apparent permeation rate of cholesterol across Caco-2 cells followed the protocol described in detail previously [30, 31]. To perform the transport experiments in the apical-to-basolateral (AP-BL, absorptive) direction, cholesterol micellar solutions (0.5 mL) with 2 μCi/mL [1,2-^3^H (N)]-cholesterol, 100 μmol/L cholesterol, 1 mmol/L OLA for control or 0.5 mmol/L indicated fatty acid (PAM / OLA / LNA / ARA / EPA / DHA), 0.5 mmol/L monoolein, 6.6 mmol/L sodium taurocholate, and 0.1 mmol/L soy PtdCho in HBSS were added to 12-well transwell inserts where Caco-2 cells were grown for additional 21 days. HBSS + 20 mmol/L HEPES + 4% BSA (pH 7.4, 1.5 mL) were used as the receiving medium throughout the study. BSA was at a final concentration of 4% in the basolateral chamber, which closely mimicked the physiological situation. Samples from the receiving compartment were collected after 0, 30, 60, 90 and 120 min, respectively. During the experiments, each sampling volume (100 μL) was replaced by an equal volume of blank transport medium. For the transport of the basolateral-to-apical (BL-AP, secretory) direction, cholesterol micellar solutions (1.5 mL) as the donor solution were added to 12-well transwell BL side and HBSS solutions (0.5 mL) as the receiving medium were added to transwell inserts (AP side). Samples were stored at −20 °C for liquid scintillation counting.

The cumulative quantity of the transported cholesterol was expressed as an apparent permeation rate (*P*_app_ (in cm/s)) in Eq. (2), where *dQ/dt* is the cholesterol permeation flux (dpm/s), *A* is the membrane surface area of the cell monolayer(cm^2^) and *C*_*0*_ is the initial cholesterol concentration in the donor chamber (dpm/cm^3^). The ‘cumulative fraction transported’ FAcum is defined as in Eq. (3), where *t*_*i*_ is the time point for the sampling occasion *i*, *t*_*k*_ is the time point for the sampling occasion *k*, the variables *C* and *V* denote the concentration and volume in the donor (index D) or receiver (index R), respectively, *f* = *1* – V_S_*/V*_*R*_ is the sample replacement dilution factor, and V_S_ is the sample volume. When the receiver concentration exceeds 10% of the donor concentration, *P*_app_ is determined by nonlinear curve fitting as Eq. (4), where *M* is the total amount of substance in the system, *C*_*R0*_ is the concentration of the substance in the receiver compartment at the start of the time interval and *C*_*R*_*(t)* is the concentration of the substance at the time *t* measured from the start of the time interval. The uptake ratio is defined as the quotient of the absorptive permeability and the secretory permeability (*P*_app_, _AP-BL_/*P*_app_, _BL-AP_).

2 $$ {P}_{app}=\left(\frac{dQ}{dt}\right)\times \left(\frac{1}{AC_0}\right) $$

3 $$ \mathrm{FAcum}=\frac{1}{A}\sum \limits_{k=1}^i\frac{\left[{C}_R\left({t}_K\right)-f\times {C}_R\left({t}_{K-1}\right)\right]\times {V}_R}{\left[{C}_D\left({t}_{K-1}\right)+{C}_D\left({t}_K\right)\right]/2\;} $$

4 $$ {C}_R\left(\mathrm{t}\right)=\left(\frac{M_{tot}}{V_D+{V}_R}\right)+{\left({C}_{R0}-\frac{M_{tot}}{V_D+{V}_R}\right)}^{\times {e}^{-{P}_{app}\times A\times \left(\frac{1}{V_R}+\frac{1}{V_D}\right)\times t}} $$

### Determination of NPC1L1 protein by western-blot

The Caco-2 cells, which had grown for 21 days, were incubated in 6-well plates (2 mL) for 24 h in cholesterol micellar cultural medium containing the delipidized FBS with fatty acids at concentration of 0 mmol/L (for control) or 0.1 / 0.5 / 1.0 mmol/L. Then whole cell lysates were obtained by repeated freezing and thawing in homogenization buffer containing PBS pH 7.4 and complete miniprotease inhibitors (Roche Diagnostics Ltd., Manheim, Germany). Samples were kept at −80 °C until the assay was performed. Total protein content was measured using the Bradford Protein Assay Kit (Beyotime, Shanghai, China). Proteins (50 μg) in cell lysate were separated in 10% SDS-PAGE gels and transferred to a PVDF membrane electrophoretically overnight. The membranes were blotted with anti-NPC1L1 antibody (1: 1000) and then with a second Goat Anti-Rabbit IgG H&L antibody (1: 10000). The specific NPC1L1 band (150 kD) was detected with Super Signal West Pico Chemiluminescent Substrate (Thermo Scientific, Rockford, USA), and analyzed using the Quantity One 4.62 software (Bio-Rad, Hercules, CA, USA). The density ratios of the detected protein and β-actin band in the same sample were calculated and used for quantification.

### Determination of mRNA expressions by real-time quantitative PCR

The Caco-2 cells were incubated in media containing fatty acids as described previously. After incubation, total RNA was isolated using TRIZOL reagent (Life Technologies Co., Carlsbad, CA, USA) according to the manufacturer’s protocol. RNA concentration was measured using a UV spectrophotometer at 260 nm and RNA quality was determined by the ratio of 260/280 nm. Total RNA was run in triplicate and subjected to RT–qPCR amplification using the One-Step RT–qPCR Quick Master Mix (Toyobo Co. Ltd., Tokyo, Japan). The expression levels of mRNA of genes were measured using Real Time PCR Master Mix (SYBR Green; Toyobo Co. Ltd., Tokyo, Japan) and an iCycler (Bio-Rad, Hercules, CA, USA). For determining mRNA levels, a threshold cycle was obtained from each amplification curve using iQ5 software (Bio-Rad, Bio-Rad, Hercules, CA, USA). Calculation of the relative mRNA concentration was performed using the 2^-△△C^_T_ threshold cycle method [39]. Primer sets for NPC1L1, ABCG5, ABCG8, ABCA1, ACAT2, MTP, Caveolin 1, Annexin A2, SREBP-1, SREBP-2 and GAPDH genes were provided by manufacturer according to our predesign and were used for real-time PCR amplifications. GAPDH was used as housekeeping genes to normalize the results of the genes of interest. The primer sets sequences used for RT–qPCR are described in Table 2. The total reaction volume was adjusted to 25 μl. Five microliter 1:100 dilution of each cDNA was used for each reaction with primers, which were all used at a final concentration of 100 nmol/L. The annealing temperature was set at 60 – 62 °C, and 40 amplification cycles were performed. Melting curves were obtained after each run and a single distinct peak was acquired for each primer set.

**Table 2:** Primer sequences used for the RT-qPCR analysis

Gene Name	Accession Number	Primer sequences (5′-3′)
GAPDH	NM_002046	F: CATGAGAAGTATGACAACAGCCT
		R: AGTCCTTCCACGATACCAAAGT
NPC1L1	NM_013389	F: ATATCTGGCCCCAATAAGGAC
		R: ATGAACCTGGAGGCTAAAACC
ABCG5	NM_022436	F: CTCTTGTGCTACTTGGTATCGTC
		R: CTGCCACAAGTGAAATTCAGTCC
ABCG8	NM_022437	F: ATCGGCTACCCCTGTCCTC
		R: GTCCTCGTCAAGATCCTTCGT
ABCA1	NM_005502	F: CTAAAAGAGAAACACCGGAAC
		R: TGTGAGAACTGCAACGTCCA
ACAT2	NM_005891	F: CAGATGAGTTTCCTCGCCAT
		R: AACTATCCGTGCTAAAGGTG
MTP	NM_000253	F: AGAATATACCACCAAAACCGTA
		R: CTTCAGAACTCGACGGACA
Caveolin 1	NM_001753	F: CTGGCAACATCTTTATCCGTA
		R: AATGTTGAGCCACTAAACCAC
Annexin A2	BC001388	F: TTGAAACAGCCATCAAGACCA
		R: TTCAATAGGCCCAAAATCACC
SREBP-1	NM_004176	F: ACCGCTCCTCCATCAATGAC
		R: CAGGCCGACACCAGATCCTTC
SREBP-2	NM_004599	F: ATCGCTCCTCCATCAATGAC
		R: TCGATGCCCTTTAGAAGCTTG

### Statistical analysis

All experiments were performed in triplicate and values are expressed as mean ± standard deviation. The statistical significance between the groups was assessed by one-way ANOVA and was calculated using the Bonferroni multiple comparison test by the SPSS 18.0 statistical software (SPSS Inc., IL, USA). Probability values of *p* < 0.05 were considered to be statistically significant.

## Results

### Quality control of Caco-2 cell monolayer

**Fig. 1:**
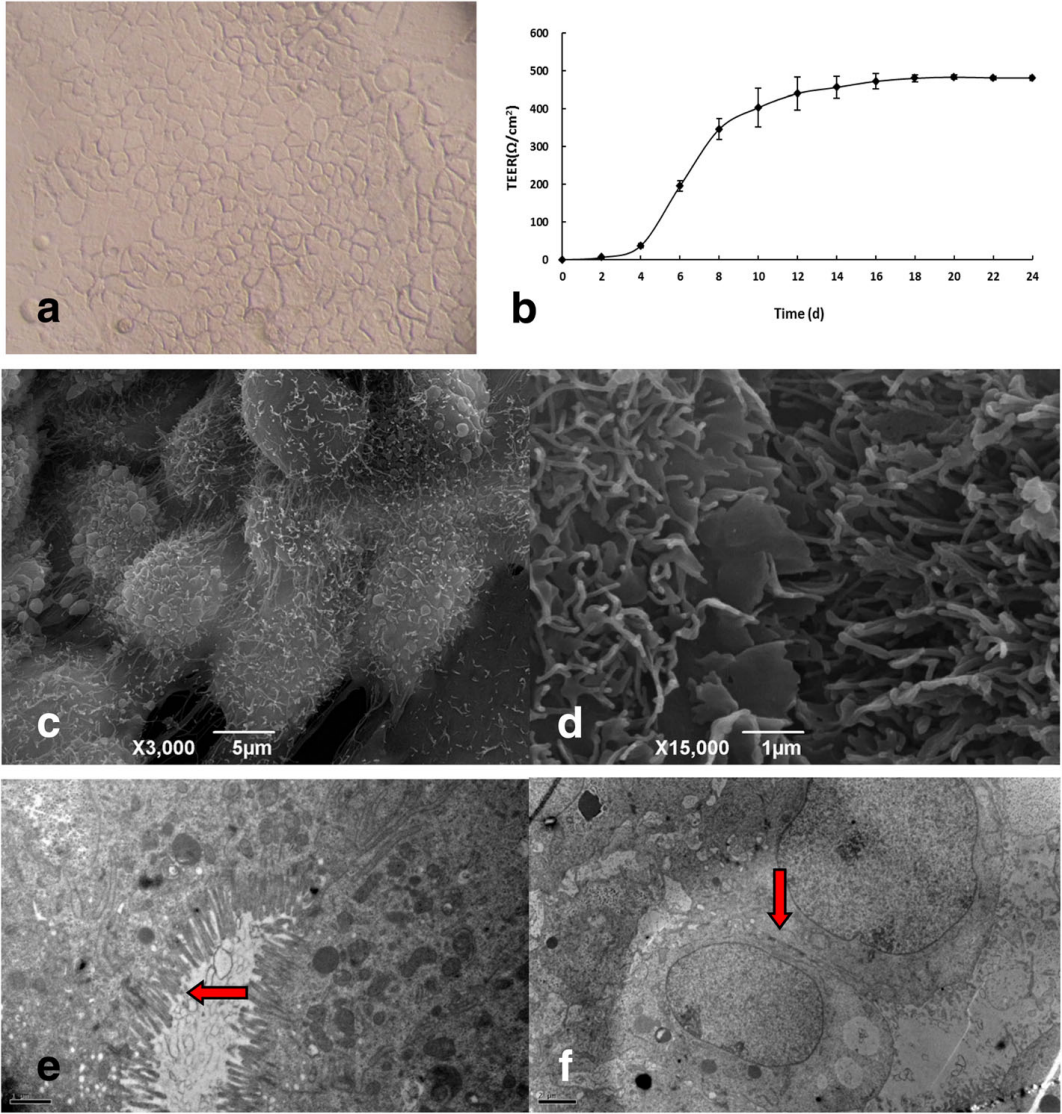
Morphology and characteristics of Caco-2 cell monolayer during and after differentiation. **a** Caco-2 monolayer morphology after the 21-day culture (Inverted microscope ×400). **b** Transepithelial electrical resistances (TEER) of Caco-2 monolayer at different time spots of the 24-day culture. The electrode of the Milicell ERS-2 resistance meter (Millipore, MA, USA) was immersed in 70% ethanol for 15 min, dried in air for 15 min, and then immersed in sterile electrolyte similar to the culture medium for 15 min. Then the two sides of electrodes were placed in two chambers of the TransWell™ culture plate, and the TEER of Caco-2 monolayer was detected, which was calculated as in Eq. (1) described in the Materials and Methods section. Results were presented as mean ± SD of three independent experiments. **c** Morphology of Caco-2 monolayer (C: × 3000) under scanning electron microscope. **d** Microvilli on the apical side of the Caco-2 monolayer (D: × 15,000) under scanning electron microscope. Cells after the 21-day culture were fixed in 2.5% glutaraldehyde treatment for 2 h, and then post-fixed for 30 min in 1% osmium tetroxide buffer and incubated in freshly made 1% carbohydrazide for 30 min. The fixed cells were rinsed three times with distilled water for over 15 min each time. The bottom of cell culture dishes was cut to fit the critical point dryer. Then, after a series of alcohol dehydration, dried in critical point dryer and sputter coat with 1-2 nm gold-palladium. Then, the cell ultrastructure was observed under scanning electron micrograph (JSM-6390/LV). **e** and **f,** the transmission electron micrographs of differentiated Caco-2 cells with microvilli (E: × 12,000) and tight junctions among cells (F: × 6000), respectively. Cells cultured for 21 days were fixed in 2.5% glutaraldehyde solution for 4 h and then post-fixed for 2 h in 1.33% osmium tetroxide buffered with 0.1 mol/L cacodylate. Then after a series of alcohol dehydration, propylene oxide/resin 2:1 infiltration, resin embedding and sectioning, the cell ultrastructure was observed under transmission electron microscopy (Hitachi H-7100)

When establishing the technique for growing Caco-2 monolayers on filter supports, it is necessary to control the morphology of the cell monolayer. The morphology is preferably evaluated by inverted micrograph (Fig. 1a), scanning electron micrograph (C and D) and transmission electron micrograph (E and F). When Caco-2 cells were cultured for 21 days, as shown in Fig. 1, those well-differentiated cells contained microvilli (D and E) and tight junctions between the cells (F) under the scanning electron micrograph and the transmission electron micrograph. The formations of Caco-2 monolayer microvilli and tight junctions among the cells were respectively observed on the apical side of the small intestinal epithelial cells, and the other side of differentiated monolayer in transwell inserts, which is equivalent to the basolateral membrane.

After 20−24 days of in vitro cell cultured, the alkaline phosphatase activity ratio in the apical side was 15.65 ± 0.79 (Table 3) and the TEER reached a relatively stable value of 517 ± 24 Ω·cm^2^ (Fig. 1b). The Lucifer Yellow apparent permeability coefficient was 2.23 × 10^−7^ cm/s which was lower than 1.0 × 10^−6^ cm/s by provision [42]. Therefore, Caco-2 cell monolayer was appropriate for use in the transport study. The permeation rate of cholesterol across Caco-2 cell monolayer was examined in both directions of AP-BL and BL-AP in donor media at pH 7.4 with micellar solutions (100 μmol/L of cholesterol), which were the best conditions according to the preliminary experiments. As shown in Table 5 control group, *P*_app_ (AP-BL) and *P*_app_ (BL-AP) of cholesterol were (26.4 ± 0.4) and (5.6 ± 0.3) × 10^−6^ cm/s, respectively. The cholesterol uptake ratio *P*_app_ (AP-BL)/*P*_app_ (BL-AP) value was calculated as 4.73, which is more than 2, a common cutoff number that suggests an active influx.

**Table 3:** Relationship between alkaline phosphatase activity and culture time of Caco-2 monolayer^a^

t/d	Ratio of alkaline phosphatase	Activity (U/g protein)
5	1.33 ± 0.04^c^	21.83 ± 2.55^b^
9	2.76 ± 0.11^c^	21.15 ± 2.14^b^
13	5.35 ± 0.34^c^	18.70 ± 1.64^b^
17	12.15 ± 0.65^b^	57.25 ± 4.46^a^
21	15.65 ± 0.79^a^	65.79 ± 6.34^a^

^a^ Alkaline phosphatase activity of the Caco-2 monolayer was measured using the Alkaline Phosphatase Activity Assay Kit (CBA-301, Cell Biolabs, Inc., CA, USA). Each value was represented as mean ± SD of three independent experiments (a > b > c > d, all *p* < 0.01)

### Evaluation of fatty acids oxidation and cytotoxicity of fatty acids on Caco-2 cell monolayer

Before the evaluation of fatty acids on the uptake and transport of cholesterol, it needs to confirm that the lipid solutions used in the experiments were not oxidized and cytotoxic to cells. The MDA concentrations and cytotoxicity of these reagents were measured (Table 4). When fresh lipid solutions were compared with oxidized fatty acid solutions, the MDA/FA values were significantly lower or barely detectable. All fatty acids used were not toxic towards the Caco-2 monolayer at any of the tested concentrations. Cholesterol transport and gene expression experiments were carried out at a range of noncytotoxic concentrations of fatty acids.

**Table 4:** Oxidation and cytotoxicity of fatty acids

	PAM	OLA	LNA	ARA	EPA	DHA	
MDA/FA	0.1 mmol/L	–	–	–	–	0.001	0.001
(μM/μM)	0.5 mmol/L	–	–	–	0.003	0.013	0.017
	1.0 mmol/L	0.007	0.012	0.024	0.027	0.032	0.038
Cell viability (%)	0.1 mmol/L	99.36	99.83	99.97	99.84	99.89	99.93
	0.5 mmol/L	99.28	99.76	99.85	99.81	99.83	99.86
	1.0 mmol/L	98.75	99.47	99.51	99.26	99.74	99.73

FA means fatty acid. “- ” means “non-detected”

### Effects of fatty acids on the uptake of cholesterol across Caco-2 cell monolayer

**Fig. 2:**
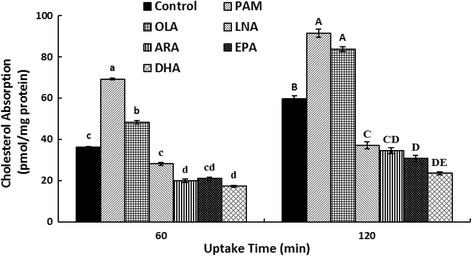
The effects of fatty acids on cholesterol uptake in Caco-2 monolayer. Caco-2 cells were incubated for 21 days, and the cholesterol uptake of 60 min and 120 min was analyzed as described under the Materials and Methods. Micellar solutions with 0.1% (*v*/v) DMSO were as follows: 2 μCi/mL [1,2-^3^H (N)]-cholesterol, 100 μmol/L cholesterol, 1 mmol/L OLA (for Control) or 0.5 mmol/L indicated fatty acid (PAM, OLA, LNA, ARA, EPA and DHA), 0.5 mmol/L monoolein, 6.6 mmol/L sodium taurocholate, and 0.1 mmol/L soy PtdCho in cultural medium containing the delipidized FBS. Data were represented as mean ± SD of three independent experiments (a > b > c > d, A > B > C > D > E, all *p* < 0.01). PAM: palmitic acid; OLA: oleic acid; LNA: linoleic acid; ARA: arachidonic acid; EPA: eicosapentaenoic acid; DHA: docosahexaenoic acid

The addition of the six fatty acids (0.5 mmol/L) in cholesterol micelles changed the uptake of micellar cholesterol in Caco-2 monolayer after the 21-day culture (Fig. 2). Compared with the control group (1 mmol/L OLA, 36.3 pmol/mg), the incorporation of micellar cholesterol into Caco-2 monolayer in the PAM and OLA groups were observably increased after the treatments for 60 min (*p* < 0.01), while decreased in ARA, EPA and DHA groups (*p* < 0.01). The addition of LNA did not influence the incorporation process. Moreover, the [^3^H]-labeled cholesterol uptake in Caco-2 monolayer of control group rose to 59.6 pmol/mg when the incubation time lasted to 120 min, the PAM and OLA groups were also significantly increased to 91.4 pmol/mg and 83.6 pmol/mg (*p* < 0.01), respectively. However, the LNA, ARA, EPA and DHA groups all significantly declined to 37.1, 34.4, 30.7 and 23.5 pmol/mg (*p* < 0.01), respectively.

### Effects of fatty acids on the transport of cholesterol across Caco-2 cell monolayer

To evaluate the accuracy of cholesterol absorption in Caco-2 monolayer, transport experiments were performed after exposure of the two side of each monolayer to cholesterol micellar solutions with fatty acids (0.5 mmol/L) in HBSS. It was shown that the effects of fatty acids on the transport of cholesterol across Caco-2 monolayer were mainly depending on the uptake (AP-BL) but not efflux (BL-AP) part in Table 5. There is no difference between the cholesterol permeation (*P*_app_, _AP-BL_) level of PAM and the control group in the AP-BL direction, while the other five fatty acids’ cholesterol permeation levels were significantly decreased compared with the control (*p* < 0.01). Compared with the control (4.73), addition of ARA, EPA and DHA significantly decreased the uptake ratio of cholesterol transport across the cell monolayer to 1.78, 1.11 and 0.86, respectively (*p* < 0.01). Additionally, cholesterol transport across the cell monolayer after 2 h of incubation was not changed by PAM, OLA and LNA.

**Table 5:** Effect of fatty acids on the transport of cholesterol across Caco-2 cell monolayer^*a*^

	*P*_app_ (×10^−6^cm/s)		Uptake Ratio	AP-BL permeation (% of control)	BL-AP permeation (% of control)
	AP-BL	BL-AP			
Control	26.4 ± 0.4^a^	5.6 ± 0.3	4.73 ± 0.2^a^	100^a^	100
PAM	18.5 ± 3.5^ab^	6.1 ± 0.8	3.05 ± 0.6^ab^	118.3 ± 16.7^a^	111.0 ± 10.3
OLA	13.7 ± 3.6^b^	6.1 ± 0.3	2.24 ± 0.5^ab^	86.8 ± 16.0^ab^	112.1 ± 10.3
LNA	13.4 ± 2.1^b^	6.2 ± 0.3	2.17 ± 0.3^ab^	85.4 ± 6.7^ab^	113.1 ± 3.4
ARA	11.0 ± 2.0^bc^	6.2 ± 0.4	1.78 ± 0.3^bc^	69.8 ± 8.0^b^	112.7 ± 1.2
EPA	6.8 ± 1.7^c^	6.1 ± 1.0	1.11 ± 0.1^c^	43.4 ± 9.0^c^	111.8 ± 11.1
DHA	5.5 ± 1.6^c^	6.4 ± 0.4	0.86 ± 0.3^c^	35.0 ± 9.0^c^	112.9 ± 17.3

^*a*^Caco-2 cells were incubated in the micellar medium after 21 days growing and micellar solutions with fatty acids were added to both the apical or basolateral sides. Micellar solutions with 0.1% (*v*/v) DMSO was as follows: 2 μCi/mL [1,2-^3^H (N)]-cholesterol, 100 μmol/L cholesterol, 1 mmol/L oleic acid (OLA for Control) or 0.5 mmol/L fatty acids (PAM, OLA, LNA, ARA, EPA and DHA), 0.5 mmol/L monoolein, 6.6 mmol/L sodium taurocholate, and 0.1 mmol/L soy PtdCho in HBSS (pH 7.4). Samples from the receiving compartment were collected at 0, 30, 60, 90 and 120 min, and analyzed by liquid scintillation counting as described under the Materials and Methods. The uptake ratio is defined as the quotient of the absorptive permeability and the secretory permeability (*P*_app_, _AP-BL_/*P*_app_, _BL-AP_). Each point represents the mean ± SD for at least three independent monolayers (a > b > c > d, all *p* < 0.01)

### Effects of fatty acids on NPC1L1 protein and mRNA expression levels in Caco-2 cells

**Fig. 3:**
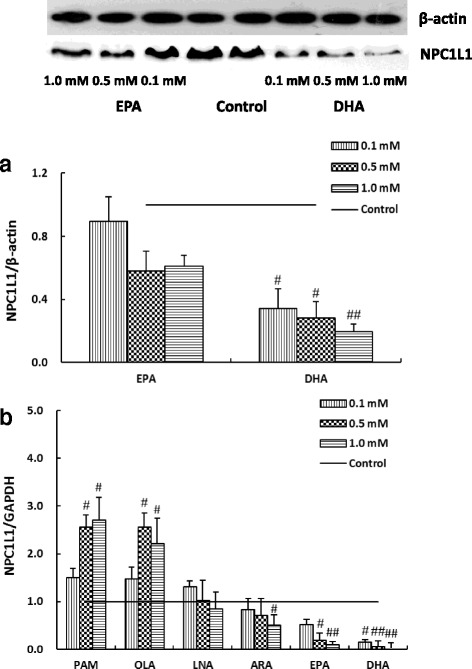
The effects of fatty acid treatments on the expression levels of Niemann-Pick C1-Like 1 (NPC1L1) protein (**a**) and its mRNA (**b**) in Caco-2 cells. Caco-2 cells were incubated in increasing concentrations of indicated fatty acids micellar solutions for 24 h, as described in the Methods and Materials section. Micellar solutions with 0.1% (v/v) DMSO were as follows: 2 μCi/mL [1,2-^3^H (N)]-cholesterol, 100 μmol/L cholesterol, 0 mmol/L (for control) or 0.1 / 0.5 / 1.0 mmol/L fatty acid (PAM, OLA, LNA, ARA, EPA or DHA), 0.5 mmol/L monoolein, 6.6 mmol/L sodium taurocholate, and 0.1 mmol/L soy PtdCho in cultural medium containing 20% delipidized FBS. Expression levels in A were normalized to that of the control β-actin protein (*P* < 0.05), and expression values in B were normalized to GAPDH mRNA, a housekeeping gene. The expression level of NPC1L1 protein or mRNA in the control group was set to 1. ^#^*p* < 0.05, ^##^*p* < 0.01 were compared to that in the control cells (Caco-2 cells incubated with micellar solution without any fatty acid). Data were presented as mean ± SD of three independent experiments. PAM: palmitic acid; OLA: oleic acid; LNA: linoleic acid; ARA: arachidonic acid; EPA: eicosapentaenoic acid; DHA: docosahexaenoic acid

To determine whether fatty acids could influence the levels of NPC1L1 in the Caco-2 cells; Caco-2 cells were incubated with increasing concentrations (0 mmol/L for control, 0.1 mmol/L, 0.5 mmol/L and 1.0 mmol/L) of these fatty acids in micellar cultural medium containing the delipidized FBS for 24 h, and the cholesterol uptake transporter NPC1L1 mRNA and protein levels were measured (Fig. 3). Compared with that of the control (value as 1, Fig. 3b), the levels of NPC1L1 mRNA in the cells treated with PAM (0.5 and 1 mmol/L) and OLA at 0.5 mmol/L and 1 mmol/L were significantly increased (*p* < 0.05) to promote cholesterol absorption, while treatment of Caco-2 enterocytes with LNA did not affect NPC1L1 mRNA expression, as normalized to that of the control gene GAPDH. However, ARA (1 mmol/L), EPA (0.5 and 1 mmol/L) and DHA dose-dependently reduced the expression of NPC1L1 mRNA (*p* < 0.05). NPC1L1 protein level in Caco-2 treated with EPA and DHA cells were shown in Fig. 3a, after band quantification and normalization with that of control protein β-actin. The protein results were different from the mRNA data. Only DHA treatment dose-dependently reduced the NPC1L1 protein levels, which were significantly decreased to 28 and 19% (*p* < 0.05) at 0.5 and 1.0 mmol/L concentrations, respectively.

### Effects of fatty acids on ABCG5 and ABCG8 mRNA expression levels in Caco-2 cells

**Fig. 4:**
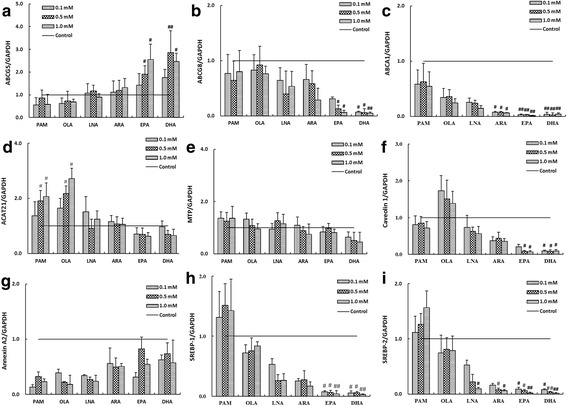
The effects of fatty acids on mRNA expression levels of ATP-binding cassette sub-family G member 5 (ABCG5) (**a**), ABCG8 (**b**), ABCA1 (**c**), acetyl-Coenzyme A acetyltransferase 2 (ACAT2) (**d**), microsomal triglyceride transfer protein (MTP) (**e**), Caveolin 1 (**f**), Annexin A2 (**g**), sterol regulatory element-binding transcription factor 1 (SREBP-1) (H) and SREBP-2 (I) in Caco-2 cells. Caco-2 cells were incubated in media with increasing concentrations of the indicated fatty acid micellar solutions for 24 h, as described in the Methods and Materials section. Micellar solutions with 0.1% (v/v) DMSO were as follows: 2 μCi/mL [1,2-^3^H (N)]-cholesterol, 100 μmol/L cholesterol, 0 mmol/L (for control) or 0.1 / 0.5 / 1.0 mmol/L fatty acids (PAM, OLA, LNA, ARA, EPA or DHA), 0.5 mmol/L monoolein, 6.6 mmol/L sodium taurocholate, and 0.1 mmol/L soy PtdCho in cultural medium containing 20% delipidized FBS. Values of the indicated genes were normalized to that of GAPDH, a housekeeping gene, which was set to 1. ^#^*p* < 0.05, ^##^*p* < 0.01 were compared to control cells (Caco-2 cells incubated with micellar solution without fatty acid). Data were represented as mean ± SD of three independent experiments. PAM: palmitic acid; OLA: oleic acid; LNA: linoleic acid; ARA: arachidonic acid; EPA: eicosapentaenoic acid; DHA: docosahexaenoic acid

The effects of fatty acids on the mRNA expressions of ABCG5 and ABCG8 in Caco-2 cells were shown in Fig. 4a and b, respectively. The results revealed that EPA and DHA at concentrations of 0.5 mmol/L and 1.0 mmol/L could significantly increased the mRNA levels of ABCG5 (*p* < 0.05), whereas the other concentrations of fatty acids did not produce any significant effect. As for the ABCG8 mRNA, EPA at 0.5 and 1.0 mmol/L and DHA decreased it markedly (*p* < 0.05).

### Effects of fatty acids on ABCA1, ACAT2 and MTP mRNA expression levels in Caco-2 cells

The addition of fatty acids also affected the mRNA level of ABCA1, a protein involved in reverse-cholesterol transport, in Caco-2 cells as shown in Fig. 4c. ABCA1 mRNA level was decreased with the incubation of ARA (*p* < 0.05), EPA (*p* < 0.01) and DHA (*p* < 0.01) at all concentrations tested, while other fatty acids did not make any difference. The mRNA levels of ACAT2 and MTP in Caco-2 cells after the treatments with fatty acids were shown in Fig. 4d and e, respectively. Compared with the control group, PAM and OLA at 0.5 mmol/L and 1.0 mmol/L significantly up-regulated the ACAT2 mRNA level (*p* < 0.05), while the other fatty acids had no significant effect on the expression levels of ACAT2 and MTP mRNA.

### Effects of fatty acids on the expression levels of Caveolin 1 and Annexin A2 mRNA in Caco-2 cells

The expression levels of Caveolin 1 and Annexin A2 mRNA in Caco-2 cells were shown in Fig. 4f and g, respectively. EPA (0.5 and 1.0 mmol/L) and DHA significantly down-regulated the mRNA level of Caveolin 1 (*p* < 0.05). Other fatty acids did not affect the expression levels of Caveolin 1 and Annexin A2 mRNA.

### Effects of fatty acids on expression levels of SREBP-1 and SREBP-2 mRNA in Caco-2 cells

Fatty acids regulated the mRNA expressions of SREBP-1 and SREBP-2 in Caco-2 cells, as shown in Fig. 4h and i, respectively. EPA and DHA at 1.0 mmol/L inhibited the expression levels of SREBP-1 mRNA by 96% (*p* < 0.01) and 98% (*p* < 0.01), respectively. ARA, EPA and DHA at 1.0 mmol/L markedly inhibited the SREBP-2 mRNA by 93% (*p* < 0.05), 98% (*p* < 0.01) and 99% (*p* < 0.01), respectively. LNA at 1.0 mmol/L inhibited the expression levels of SREBP-2 mRNA by 91% (*p* < 0.05). The inhibitory effect of PUFAs on SREBP-2 seemed to be more dramatic than that on SREBP-1.

## Discussion

Dyslipidemia is an independent and powerful risk factor for CVDs [1]. Nutritional and lifestyle modifications are the frontline for the treatment of dyslipidemia to minimize and lower CVDs risk. Current dietary recommendations focus on fatty acids and include reductions in saturated and *trans*-fatty acids, and an emphasis on consumption of mono- and poly-unsaturated fatty acids [43]. Fatty acids, which are transported in micelles with cholesterol and phospholipids, are absorbed by the enterocytes in the proximal parts of the small intestine. The effects of the different types of fatty acids on the transport of cholesterol in enterocytes have not been clearly defined. In the current study, we evaluated the effects of different fatty acids on cholesterol transport across the Caco-2 monolayer, and the possible regulatory mechanisms.

The main observation of this work is that EPA and DHA inhibited cholesterol uptake and transport in Caco-2 monolayer, which might be caused by down-regulating the expression levels of NPC1L1 mRNA and protein. However, PAM and OLA (0.5 and 1.0 mmol/L) up-regulated NPC1L1 gene expression, and therefore, increased cholesterol uptake and transport in Caco-2 monolayer. Downregulation of NPCL1L mRNA expression is associated with inhibition of the expression levels of transcription factor SREBPs. Furthermore, we have demonstrated that ARA and EPA inhibited the expression of ABCA1 to block the cholesterol efflux into the circulation via the function of HDL. EPA at high concentrations and DHA at all concentrations inhibited the mRNA expression of Caveolin 1, suggesting that EPA and DHA inhibited cholesterol uptake, and transport in Caco-2 monolayer might also be related with the expression of Caveolin 1 gene (Additional file 1: Figure S1).

Methods for the measurement of intestinal cholesterol absorption have been mainly focused on the studies of animals and humans using direct (mesenteric or thoracic lymph duct) [44, 45], indirect (fecal and plasma dual-isotope ratio) [46, 47] and the sterol balance [48] approaches. On the other hand, in vitro drug transport studies in epithelial cell monolayer now are frequently used as screening tools in drug discovery programs for the prediction of intestinal drug permeability, on the premise that a single layer of epithelial cells covers the inner intestinal wall and forms the rate-limiting barrier. The human colon carcinoma Caco-2 cell line has been found to serve this purpose well and became the golden standard for in vitro prediction of intestinal drug permeability and absorption [35, 37]. We had cultured Caco-2 cells on permeable filters after 21 days, which slowly differentiated into monolayers with a differentiated phenotype and many functions of the small intestinal villus epithelium. This allowed us to complete the quality control of Caco-2 monolayer and cholesterol permeability determination. Generally speaking, *P*_app_ > 1.0 × 10^− 6^ cm/s indicates high permeation, whereas *P*_app_ < 1.0 × 10^− 7^ cm/s implies low permeation [49]. The bidirectional transport data (Table 5, control group) for cholesterol showed that the absorptive *P*_app_ was at least four-fold higher than its secretory *P*_app_, suggesting that the uptake of cholesterol was an active influx and it may limit the absorption of cholesterol by inhibiting some key intestinal cholesterol absorption proteins.

Circulating cholesterol carried in LDL is derived primarily from absorption of the dietary cholesterol and from de novo synthesized cholesterol in tissues and organs such as the liver. In humans, there is a significant and positive correlation between the level of plasma LDL cholesterol and the efficiency of intestinal cholesterol absorption [50]. Thus, the restriction of dietary calories, cholesterol, and saturated fat has been recommended as the primary initial therapeutic intervention for the treatment of patients with dyslipidemia and CVDs [51]. Our results showed that the cholesterol uptake and transport in Caco-2 cell monolayer was gradually inhibited with the increasing chain length and unsaturation of the fatty acids (Fig. 2 and Table 5), especially for ARA, EPA and DHA groups (*p* < 0.01). This supports the findings of the previous studies that the cholesterol absorption in animal models was decreased by PUFAs when a high amount of cholesterol is present in the diet [16–19]. The results of transport experiments showed that the efficiency of cholesterol absorption may be determined by the net influx and efflux of intraluminal cholesterol molecule crossing the brush border membrane of the enterocytes.

The NPC1L1 protein, a newly identified sterol influx transporter, may actively facilitate the uptake of cholesterol by promoting the passage of sterols across the brush border membrane of the enterocytes [20]. Moreover, it has been demonstrated that human NPC1L1 expression and promoter activity are modulated by cholesterol via a SREBP-dependent mechanism [22]. SREBPs are transcription factors that bind to the sterol regulatory element, and regulate the expression levels of genes related to lipid and cholesterol production in response to the cellular sterol levels [23, 52]. On the other hand, ABCG5 and ABCG8 promote active efflux of cholesterol and plant sterols from the enterocytes into the intestinal lumen for excretion. The combined effects of NPC1L1, ABCG5, and ABCG8 may play a critical role in modulating the amount of cholesterol that reaches the lymph from the intestinal lumen eventually. In this paper, EPA and DHA could considerably down-regulate NPC1L1 gene expression, which may lead to a decrease of NPC1L1 protein level, while the cells treated with PAM and OLA at higher concentrations may have the opposite results (Fig. 3). Our results also show that the modulations of differential EPA and DHA concentrations on the expressions of NPC1L1 and SREBPs were consistent with the results of cholesterol uptake and transport, which is consistent with the in vitro study of PUFAs in Caco-2 cells, that significantly down-regulated the expression of the key intestinal cholesterol absorption protein NPC1L1 to decreased cholesterol absorption [27, 28]. However, the mechanisms by which SREBP mediated NPC1L1 expression need further study.

Several proteins involved in other steps in the absorption process may exert influences on cholesterol absorption, such as ABCA1, ACAT2 and MTP, which involve esterification of cholesterol and its incorporation into nascent chylomicrons that are subsequently secreted into the lymph [22]. These intracellular events may also exert major influences on cholesterol absorption. ARA, EPA and DHA decreased ABCA1 mRNA levels (Fig. 4c, *p* < 0.05), suggesting that these PUFAs might also have reduced the removal of cholesterol from Caco-2 cell by a mechanism similar to the reverse cholesterol transport process. Compared with the control group, high levels of PAM and OLA could significantly up-regulate the ACAT2 mRNA level (Fig. 4d, *p* < 0.05), indicating that saturated fatty acids and monounsaturated fatty acids at certain concentrations could promote cholesterol esterification and secretion in Caco-2 cells in the form of chylomicrons [53]. These findings strongly support the concept that cholesterol absorption is a multistep process regulated by multiple genes in enterocytes, and that the chain length and unsaturation of the fatty acids could affect the efficiency of cholesterol absorption determined by influx and efflux of intraluminal cholesterol molecule crossing the brush border membrane of enterocytes.

In addition, Caveolin 1 acts as a chaperone complex via the cytoplasm or by regulating cholesterol influx or efflux via plasma membrane caveolae [54], and Annexin A2 acts as a calcium-dependent phospholipid-binding protein in enterocytes [55]. EPA and DHA might inhibit cholesterol absorption by down-regulating the mRNA expression of caveolin 1 (Fig. 4f, *p* < 0.05). However, a number of cell culture studies have provided partially contradictory evidences for the role of caveolin-1 in cellular cholesterol trafficking [54]. Studies up to now are limited to the most direct and pathophysiologically relevant connection between caveolin and cholesterol transport. The regulatory effects of fatty acids on the intestinal cholesterol absorption are mainly attributed to the regulated expression levels of those membrane transporters.

## Conclusions

Here, a Caco-2 monolayer model was used to investigate the effects of fatty acids on cholesterol transport, and the possible regulation mechanisms. The current findings indicate that dietary PUFAs, which are considered as functional food ingredients, significantly inhibit the intestinal absorption of cholesterol in Caco-2 monolayer. The reason may be due to the decreased expression levels of the NPC1L1 mRNA and protein, which might be related to the decrease of SREBP-1/− 2 expressions. Findings from this study might lend support to the use of functional food containing high PUFAs with potential therapeutic benefit. All these may facilitate further study on the fatty acids and provide an individualized approach to prevent disease and promote health.

## Additional file

Additional file 1: **Figure S1.** Intestinal cholesterol absorption modulated by fatty acids is a multistep process that is regulated by multiple genes. In the lumen of the small intestine, cholesterol esters from dietary intake and biliary secretion are solubilized in mixed micelles, which containing bile acids, fatty acids and phospholipids. Then the cholesterol esters are converted to free cholesterol by cholesterol esterase before free cholesterol then across the unstirred water layer and enter into enterocytes [22]. NPC1L1 protein as a sterol transporter mediates intestinal cholesterol absorption from intestinal brush border membranes into enterocytes [20], which might be inhibited by high concentration of EPA and DHA, and induced by PAM and OLA (0.5 and 1 mM), and therefore inhibited cholesterol uptake and transport in Caco-2 monolayer. Downregulation of NPCL1L mRNA expression is associated with inhibition of transcription factor SREBP-1/− 2 [22, 23, 52]. The majority of absorbed and endogenously synthesized cholesterol is transported to the endoplasmic reticulum, where it is converted to cholesterol ester by ACAT2 and is then assembled into chylomicrons in a MTP-dependent manner for secretion into the circulation via the lymphatic system [22]. Unesterified cholesterol may be transported back to the intestinal lumen by the apically localized heterodimeric sterol transporter ABCG8 [21, 22], which might be increased by high concentrations of EPA and DHA. Cholesterol may also be transported into the circulation as a constituent of HDL via localized ABCA1 at the basolateral membrane of enterocytes, which demonstrated that ARA, EPA and DHA inhibited the expression of ABCA1 to block the cholesterol efflux into the circulation as a function of HDL. (DOC 42 kb)

## Abbreviations

ABCA1: ATP-binding cassette sub-family A member 1
ABCG5: ATP-binding cassette sub-family G member 5
ABCG8: ATP-binding cassette sub-family G member 8
ACAT2: Acetyl-Coenzyme A acetyltransferase 2
AP: Apical
ARA: Arachidonic acid
BL: Basolateral
BSA: Bovine serum albumin
cpm: Counts per minute
CVDs: Cardiovascular diseases
DHA: Docosahexaenoic acid
DMEM: Dulbecco’s modified eagle medium
DMSO: Dimethyl sulfoxide
dpm: Decay per minute
EDTA: Ethylenediaminetetraacetic acid
EPA: Eicosapentaenoic acid
FBS: Fetal bovine serum
HBSS: Hank’s balanced salt solution
HDL: High-density lipoprotein
HEPES: 4-(2-hydroxyethyl)-1-piperazineethanesulfonic acid
LDL: Low-density lipoprotein
LNA: Linoleic acid
MDA: Malondialdehyde
MTP: Microsomal triglyceride transfer protein
MTT: 3-(4, 5-dimethylthiazole-2-yl)-2, 5-diphenyltetrazolium bromide
MUFAs: Monounsaturated fatty acids
NPC1L1: Niemann-Pick C1-Like 1 protein
OLA: Oleic acid
PAM: Palmitic acid
*P*_app_: Apparent permeation rate
Popop: Para-phenylene-phenyloxazole
PPO: 2,5-diphenyloxazole
PtdCho: Phospatidylcholine
PUFAs: Polyunsaturated fatty acids
SFAs: Saturated fatty acids
SREBP-1: Sterol regulatory element-binding transcription factor 1
SREBP-2: Sterol regulatory element-binding transcription factor 2
TEER: Transepithelial electrical resistance
