# Supplementary material for: Fatty acids modulate the expression levels of key proteins for cholesterol absorption in Caco-2 monolayer
Source: Lipids Health Dis. 2018 Feb 20;17:32. doi: 10.1186/s12944-018-0675-y (PMC5819267; doi:10.1186/s12944-018-0675-y)
Supplement: Supplementary file 1 — Figure S1. Intestinal cholesterol absorption modulated by fatty acids is a multistep process that is regulated by multiple genes. In the lumen of the small intestine, cholesterol esters from dietary intake and biliary secretion are solubilized in mixed micelles, which containing bile acids, fatty acids and phospholipids. Then the cholesterol esters are converted to free cholesterol by cholesterol esterase before free cholesterol then across the unstirred water layer and enter into enterocytes [22]. NPC1L1 protein as a sterol transporter mediates intestinal cholesterol absorption from intestinal brush border membranes into enterocytes [20], which might be inhibited by high concentration of EPA and DHA, and induced by PAM and OLA (0.5 and 1 mM), and therefore inhibited cholesterol uptake and transport in Caco-2 monolayer. Downregulation of NPCL1L mRNA expression is associated with inhibition of transcription factor SREBP-1/− 2 [22, 23, 52]. The majority of absorbed and endogenously synthesized cholesterol is transported to the endoplasmic reticulum, where it is converted to cholesterol ester by ACAT2 and is then assembled into chylomicrons in a MTP-dependent manner for secretion into the circulation via the lymphatic system [22]. Unesterified cholesterol may be transported back to the intestinal lumen by the apically localized heterodimeric sterol transporter ABCG8 [21, 22], which might be increased by high concentrations of EPA and DHA. Cholesterol may also be transported into the circulation as a constituent of HDL via localized ABCA1 at the basolateral membrane of enterocytes, which demonstrated that ARA, EPA and DHA inhibited the expression of ABCA1 to block the cholesterol efflux into the circulation as a function of HDL. (DOC 42 kb) [file 12944_2018_675_MOESM1_ESM.doc]

**FAs with cholesteryl esters**

**NPC1L1**

**SREBP-1**

**Cholesterol**

**Nascent HDL**

**ACAT2**

**MTP**

**ABCA1**

**APO-A1**

**Cholesterol esters**

**Lumen**

**Enterocyteer**

**Lymph**

**Cholesterol**

**Annexin**

**SREBP-2**

**EPA and DHA**

**ARA, EPA and DHA**

**ARA, EPA**

**and DHA**

**Cholesteryl esters**

**PAM (0.5 and 1 mM)**

**OLA (0.5 and 1 mM)**

**EPA (0.5 and 1 mM)**

**DHA**

**PAM (0.5 and 1 mM)**

**OLA (0.5 and 1 mM)**

**EPA (0.5 and 1.0 mM)**

**DHA (0.5 and 1.0 mM)**

**Fatty acids**

**Cholesterol absorption**

**Cholesterol esterase**

**Mixed micelle**

**Cholesterol**

**APO-B48**

**Caveolin 1**

**ABCG5**

**ABCG8**

**Chylomicrons**
